# Cardiovascular Health, Functional Disability, and Leisure Activities Profiles in Relation to Mental Aging

**DOI:** 10.1093/geroni/igaa057.2292

**Published:** 2020-12-16

**Authors:** Dorina Cadar, Celine De Looze, Christine McGarrigle

## Abstract

We investigated cardiovascular health, functional disability and leisure activities profiles independently and in relation to cognitive decline and dementia in high and low-medium income countries using data from the English Longitudinal Study of Ageing, Irish Longitudinal Study on Ageing and Brazilian Bambui Cohort Study of Aging. Functional loss among older Brazilians has shown a hierarchical sequence over the 15-year follow-up, with the highest incidence in functional disability reported for dressing, followed by getting out of bed, bathing/showering, walking across a room, using the toilet and eating (de Oliveira). Using the Life’s Simple 7, an ideal cardiovascular health scoring system evaluating the muscular strength, mobility and physiological stress, we showed a reliable prediction of cognitive trajectories in a representative sample of Irish individuals (De Looze). Within the same cohort, we report discrepancies between men and women in functional decline driven by domestic tasks, rather than determining differential cognitive trajectories (McGarrigle). In an English representative sample, we found that participants with an increasing number of functional impairments over almost a decade were more likely to be classified with subsequent dementia compared with those with no impairments and this may imply a more comprehensive ascertainment during the prodromal stage of dementia (Cadar). In contrast, a reduced risk of dementia was found for individuals with higher levels of engagement in cognitively stimulating activities, that may preserve cognitive reserve until later in life (Almeida). Identifying factors that influence cognitive aging and dementia risk in a multifactorial perspective is critical toward developing adequate intervention and treatment.

